# Supplementary material for: Niclosamide nanoparticles as a novel adjuvant reverse colistin resistance via multiple mechanisms against multidrug-resistant Salmonella infections
Source: Microbiol Spectr. 2025 Oct 20;13(12):e02252-25. doi: 10.1128/spectrum.02252-25 (PMC12671199; doi:10.1128/spectrum.02252-25)
Supplement: Figures S1 to S5 and Tables S1 to S3. — Niclosamide nanoparticles as a novel adjuvant reverse colistin resistance via multiple mechanisms against multidrug-resistant Salmonella infections. [file spectrum.02252-25-s0001.docx]

**Supplemental material**

**Niclosamide nanoparticles as a novel adjuvant reverse colistin resistance via multiple mechanisms against multidrug-resistant *Salmonella* infections**

Kaifang Yi^1,2^, Peiyi Liu^1,2^, Mengyao Zhang^1,2^, Qiange Liu^1,2^, Mengjing Feng^1,2^, Zibo Li^3^, Dandan He^1,2^, Li Yuan^1,2^, Xiaoyuan Ma^1,2^* and Gongzheng Hu^1,2^*,

^1^Henan Agricultural University, Zhengzhou, China

^2^Ministry of Education Key Laboratory for Animal Pathogens and Biosafety, Zhengzhou, China

^3^Shangqiu Meilan Biological Engineering Co., LTD, Shangqiu, Henan, China.

*Corresponding author: Xiaoyuan Ma, E-mail: lisamxy@126.com; Gongzheng Hu, E-mail: yaolilab@126.com

**Supplementary Figure**


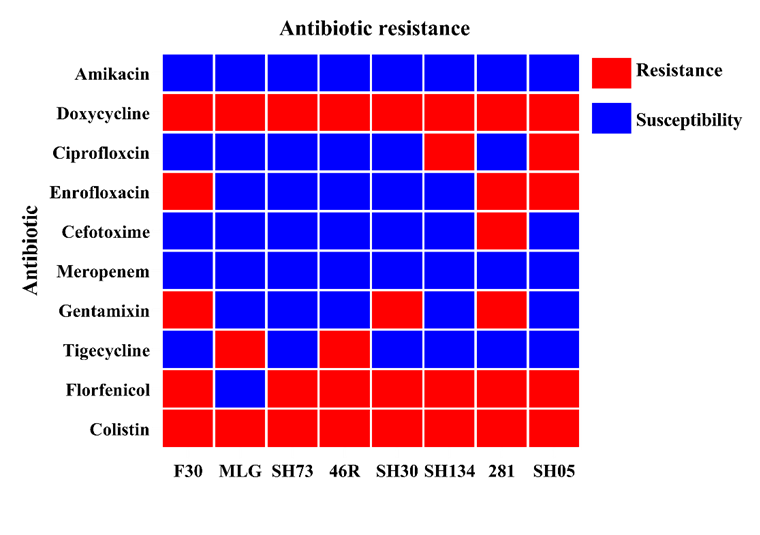


**Fig. S1**. Resistance spectrum of 8 clinical *Salmonella* strains.


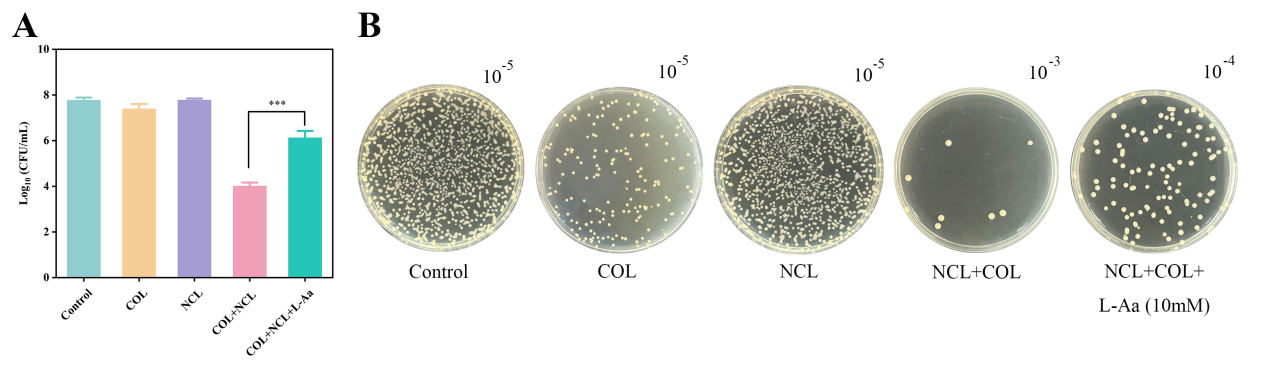


**Fig. S2**. (A) Counts of bacterial colony forming units of *mcr*-1-carrying *Salmonella* SH05 after niclosamide in combination with colistin with or without ROS quencher L-Ascorbic acid (L-Aa) (n = 3; (B) Plate colony images of bacteria treated with niclosamide in combination with colistin in the presence or absence of ROS quencher L-ascorbic acid (L-Aa, 10 mM). Statistical analysis was conducted using student’s t-test (****p* < 0.001).

**
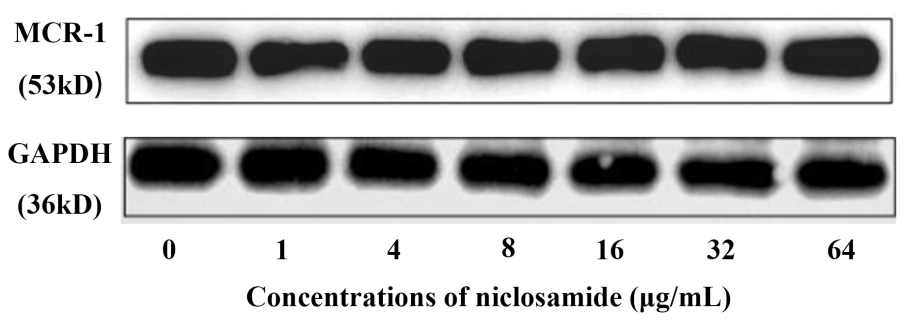
**

**Fig. S3**. Western blot analysis showed the expression of MCR-1 after treatment with niclosamide at different concentrations.


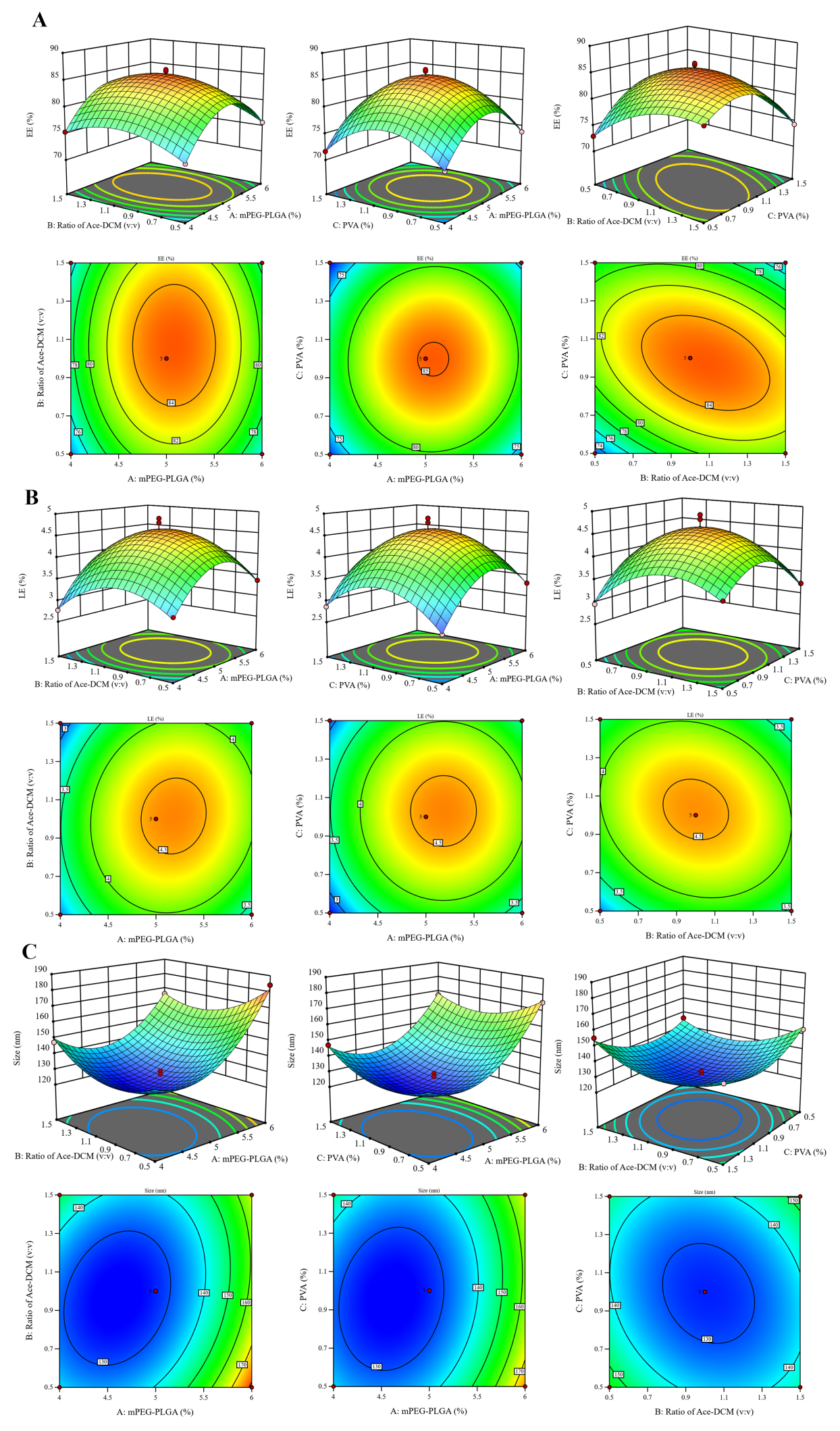


**Fig. S4**. Preparation of NCL@mPEG-PLGA-NPs. The response surface model demonstrated the influence of mPEG-PLGA concentration, volume ratio of dichloromethane to acetone, and PVA concentration on nanoparticles NIC-EE (A), COL-EE (B), and size (C).


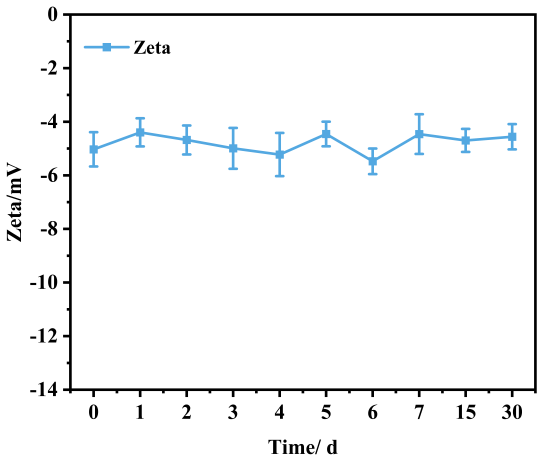


**Fig. S5**. Changes in the zeta potential of NCL@mPEG-PLGA-NPs stored in PBS media at 4℃ for 30 days.

**Supplementary Tables**

**Table S1. Strains of bacteria used in this study**

| Bacteria strains | *mcr*-1 | Source |
| --- | --- | --- |
| JS | negative | Standard strains |
| 15R | negative | chicken |
| 40R | negative | chicken |
| 46R | negative | chicken |
| 53R | negative | chicken |
| SH73 | positive | chicken |
| F232 | positive | chicken |
| YLG | negative | chicken |
| LEG | negative | chicken |
| SH05 | positive | chicken |
| S506 | negative | chicken |
| SH22 | positive | chicken |
| SH30 | positive | chicken |
| SH13 | positive | chicken |
| S2 | positive | chicken |
| S57 | negative | chicken |
| F30 | positive | chicken |
| S28 | positive | chicken |
| F162 | positive | chicken |
| S60s | negative | chicken |

**Table S2. Sequences of primers used in this study**

| Primer | Sequence （5′ → 3′） |
| --- | --- |
| *16S rRNA-*qF | CCTCAGCACATTGACGTTAC |
| *16S rRNA*-qR | TTCCTCCAGATCTCTACGCA |
| *mcr-1*-qF | ACACTTATGGCACGGTCTATG |
| *mcr-1*-qR | GCACACCCAAACCAATGATAC |

**Table S3. Resistance reversal index of different concentrations of niclosamide against 8 isolates of wild-type *Salmonella***

| Strains | C_(NCL)_ ^a^ | 0.016 | 0.031 | 0.03 | 0.125 | 0.25 | 0.5 | 1 | 2 | 4 | 8 | 16 | 32 | 64 | 128 | 256 |
| --- | --- | --- | --- | --- | --- | --- | --- | --- | --- | --- | --- | --- | --- | --- | --- | --- |
| SH05 | MIC_(COL)_^b^ | — | — | — | — | 2 | 1 | 0.25 | 0.25 | 0.25 | 0.25 | 0.25 | 1 | 1 | 1 | 1 |
|  | RF^c^ | — | — | — | — | 1 | 2 | 8 | 8 | 8 | 8 | 8 | 2 | 2 | 2 | 2 |
|  | IF^d^ | — | — | — | — | 1 | 2 | 4 | 8 | 16 | 32 | 64 | 128 | 256 | 512 | 1024 |
|  | RRI^e^ | — | — | — | — | 1 | 1 | 2 | 1 | 0.5 | 0.25 | 0.125 | 0.016 | 0.008 | 0.004 | 0.002 |
|  | CR^f^ | — | — | — | — | 1/8 | 1/2 | 4/1 | 8/1 | 16/1 | 32/1 | 64/1 | 32/1 | 64/1 | 128/1 | 256/1 |
| YLG | MIC_(COL)_ | — | — | — | — | — | 2 | 0.5 | 0.5 | 0.5 | 0.5 | 0.5 | 0.5 | 1 | 2 | 2 |
|  | RF | — | — | — | — | — | 1 | 4 | 4 | 4 | 4 | 4 | 4 | 2 | 1 | 1 |
|  | IF | — | — | — | — | — | 1 | 2 | 4 | 8 | 16 | 32 | 64 | 128 | 256 | 512 |
|  | RRI | — | — | — | — | — | 1 | 2 | 1 | 0.5 | 0.25 | 0.125 | 0.063 | 0.016 | 0.004 | 0.002 |
|  | CR | — | — | — | — | — | 1/4 | 2/1 | 4/1 | 8/1 | 16/1 | 32/1 | 64/1 | 64/1 | 64/1 | 128/1 |
| F30 | MIC_(COL)_ | — | — | — | 2 | 1 | 0.5 | 0.25 | 0.25 | 0.25 | 0.25 | 0.25 | 0.25 | 1 | 2 | 2 |
|  | RF | — | — | — | 1 | 2 | 4 | 8 | 8 | 8 | 8 | 8 | 8 | 2 | 1 | 1 |
|  | IF | — | — | — | 1 | 2 | 4 | 8 | 16 | 32 | 64 | 128 | 256 | 512 | 1024 | 2048 |
|  | RRI | — | — | — | 1 | 1 | 1 | 1 | 0.5 | 0.25 | 0.125 | 0.063 | 0.031 | 0.004 | 0.001 | 0.0005 |
|  | CR | — | — | — | 1/16 | 1/4 | 1/1 | 4/1 | 8/1 | 16/1 | 32/1 | 64/1 | 128/1 | 64/1 | 64/1 | 128/1 |
| SH30 | MIC_(COL)_ | — | — | — | 2 | 1 | 0.5 | 0.25 | 0.25 | 0.25 | 0.25 | 0.25 | 1 | 1 | 2 | 2 |
|  | RF | — | — | — | 1 | 2 | 4 | 8 | 8 | 8 | 8 | 8 | 2 | 2 | 1 | 1 |
|  | IF | — | — | — | 1 | 2 | 4 | 8 | 16 | 32 | 64 | 128 | 256 | 512 | 1024 | 2048 |
|  | RRI | — | — | — | 1 | 1 | 1 | 1 | 0. 5 | 0.25 | 0.125 | 0.063 | 0.008 | 0.004 | 0.001 | 0.0005 |
|  | CR | — | — | — | 1/16 | 1/4 | 1/1 | 4/1 | 8/1 | 16/1 | 32/1 | 64/1 | 32/1 | 64/1 | 64/1 | 128/1 |
| SH73 | MIC_(COL)_ | — | — | — | — | 2 | 0.5 | 0.25 | 0.25 | 0.25 | 0.25 | 0.5 | 0.5 | 0.5 | 2 | 2 |
|  | RF | — | — | — | — | 1 | 4 | 8 | 8 | 8 | 8 | 4 | 4 | 4 | 1 | 1 |
|  | IF | — | — | — | — | 1 | 2 | 4 | 8 | 16 | 32 | 64 | 128 | 256 | 512 | 1024 |
|  | RRI | — | — | — | — | 1 | 2 | 2 | 1 | 0.5 | 0.25 | 0.063 | 0.031 | 0.016 | 0.002 | 0.001 |
|  | CR | — | — | — | — | 1/8 | 1/1 | 4/1 | 8/1 | 16/1 | 32/1 | 32/1 | 64/1 | 128/1 | 64/1 | 128/1 |
| SH13 | MIC_(COL)_ | — | — | — | 2 | 1 | 0.5 | 0.25 | 0.25 | 0.25 | 0.25 | 0.25 | 0.25 | 1 | 2 | 2 |
|  | RF | — | — | — | 1 | 2 | 4 | 8 | 8 | 8 | 8 | 8 | 8 | 2 | 1 | 1 |
|  | IF | — | — | — | 1 | 2 | 4 | 8 | 16 | 32 | 64 | 128 | 256 | 512 | 1024 | 2048 |
|  | RRI | — | — | — | 1 | 1 | 1 | 1 | 0.5 | 0.25 | 0.125 | 0.063 | 0.031 | 0.004 | 0.001 | 0.0005 |
|  | CR | — | — | — | 1/16 | 1/4 | 1/1 | 4/1 | 8/1 | 16/1 | 32/1 | 64/1 | 128/1 | 64/1 | 64/1 | 128/1 |
| S28 | MIC_(COL)_ | — | — | — | 2 | 2 | 0.5 | 0.25 | 0.25 | 0.25 | 0.25 | 0.5 | 0.5 | 1 | 2 | 2 |
|  | RF | — | — | — | 1 | 1 | 4 | 8 | 8 | 8 | 8 | 4 | 4 | 2 | 1 | 1 |
|  | IF | — | — | — | 1 | 2 | 4 | 8 | 16 | 32 | 64 | 128 | 256 | 512 | 1024 | 2048 |
|  | RRI | — | — | — | 1 | 0.5 | 1 | 1 | 0.5 | 0.25 | 0.125 | 0.031 | 0.016 | 0.004 | 0.001 | 0.0005 |
|  | CR | — | — | — | 1/16 | 1/8 | 1/1 | 4/1 | 8/1 | 16/1 | 32/1 | 32/1 | 64/1 | 64/1 | 64/1 | 128/1 |
| 46R | MIC_(COL)_ | 2 | 0.5 | 0.25 | 0.25 | 0.125 | 0.063 | 0.031 | 0.031 | 0.031 | 0.031 | 0.063 | 0.125 | 0.125 | 1 | 1 |
|  | RF | 1 | 4 | 8 | 8 | 16 | 32 | 64 | 64 | 64 | 64 | 32 | 16 | 16 | 2 | 2 |
|  | IF | 1 | 2 | 4 | 8 | 16 | 32 | 64 | 128 | 256 | 512 | 1024 | 2048 | 4096 | 8192 | 16384 |
|  | RRI | 1 | 2 | 2 | 1 | 1 | 1 | 1 | 0.5 | 0.25 | 0.125 | 0.031 | 0.008 | 0.004 | 0.0002 | 0.0001 |
|  | CR | 1/128 | 1/16 | 1/4 | 1/2 | 2/1 | 8/1 | 32/1 | 64/1 | 128/1 | 256/1 | 256/1 | 256/1 | 512/1 | 128/1 | 256/1 |

^a^ MIC_(COL)_ is the MIC of colistin in the presence of a corresponding concentration of niclosamide.

^b^ C_(NCL)_ is niclosamide concentration (μg/mL).

^c^ The reversal fold of colistin resistance reversal (Reversal fold, RF) was calculated by dividing the susceptibility breakpoint of colistin (2 μg/mL) by the MIC of colistin in the presence of niclosamide.

^d^ the increased fold in the MRC of niclosamide (Increase fold, IF) was calculated by dividing the concentration of niclosamide by the MRC of niclosamide.

^e^ RRI is resistance reversal index of niclosamide.

^f^ The concentration ratio of adjuvant to colistin was calculated by dividing the concentration of niclosamide by the MIC of colistin in the presence of niclosamide.

“—” the means is no data was read.
